# JAK2 V617F mutation, multiple hematologic and non-hematologic processes: an association?

**DOI:** 10.1186/s40364-016-0073-4

**Published:** 2016-10-19

**Authors:** Kenneth G. Liu, Amit Verma, Olga Derman, Noah Kornblum, Murali Janakiram, Ira Braunschweig, Ramakrishna Battini

**Affiliations:** Department of Medical Oncology, Montefiore Medical Center, Bronx, NY 10467 USA

**Keywords:** Case report, Lung adenocarcinoma, Myeloproliferative neoplasm, Chronic myelomonocytic leukemia, Diffuse large B-cell lymphoma, Acute myeloid leukemia, JAK2 V617F

## Abstract

**Background:**

Population studies showed that patients with JAK2 V617F mutation had increased mortality, and increased risk of any cancer, hematologic cancer, and myeloproliferative disease.

**Case presentation:**

A 68-year-old Asian male with JAK2 V617F mutation developed four different hematologic and non-hematologic neoplastic processes. In 2009, he was diagnosed with stage IA lung adenocarcinoma and also noted to have worsening leukocytosis and thrombocytosis with peak platelet count of 1,054,000/mL). Bone marrow biopsy was consistent with myeloproliferative neoplasm. His monocyte percentage increased in 2011 and met criteria for chronic myelomonocytic leukemia. In 2013, he was admitted for proximal small bowel obstruction, with biopsy confirming stage IE diffuse large B-cell lymphoma. In 2014, a bone marrow biopsy performed for worsening leukocytosis was consistent with acute myeloid leukemia with monocytic differentiation.

**Conclusion:**

This is a rare case depicting the association of JAK2 V617F mutation with myeloproliferative, lymphoproliferative and solid neoplasms.

## Background

Myeloproliferative neoplasm (MPN) and chronic myelomonocytic leukemia (CMML) have the potential to progress into acute myeloid leukemia (AML), and some data also suggests an association between myeloproliferative and lymphoproliferative neoplasms (LPN's). In this article, we describe a patient with JAK2 V617F mutation who developed 4 different neoplastic processes: adenocarcinoma of the lung, MPN/CMML, diffuse large B-cell lymphoma (DLBCL), acute myeloid leukemia with monocytic differentiation (AML-M5). The patient also had a translocation of chromosome 4 and 11, which is only seen in a few case reports in therapy-related AML's following exposure to topoisomerase II inhibitors [1]. He received doxorubicin, a topoisomerase II inhibitor, as part of chemotherapy regimen for his DLBCL approximately 6-9 months prior to the diagnosis of AML-M5. What is even more intriguing about this case is the fact that the patient had a JAK2 V617F mutation, which has been reported to be associated with an increased risk of cancer and mortality [2]. The coexistence of four hematologic and non-hematologic disorders in the same patient with JAK2 V617F mutation is very rare, and we review here the literature on the association of these processes.

## Case presentation

**Fig. 1:**
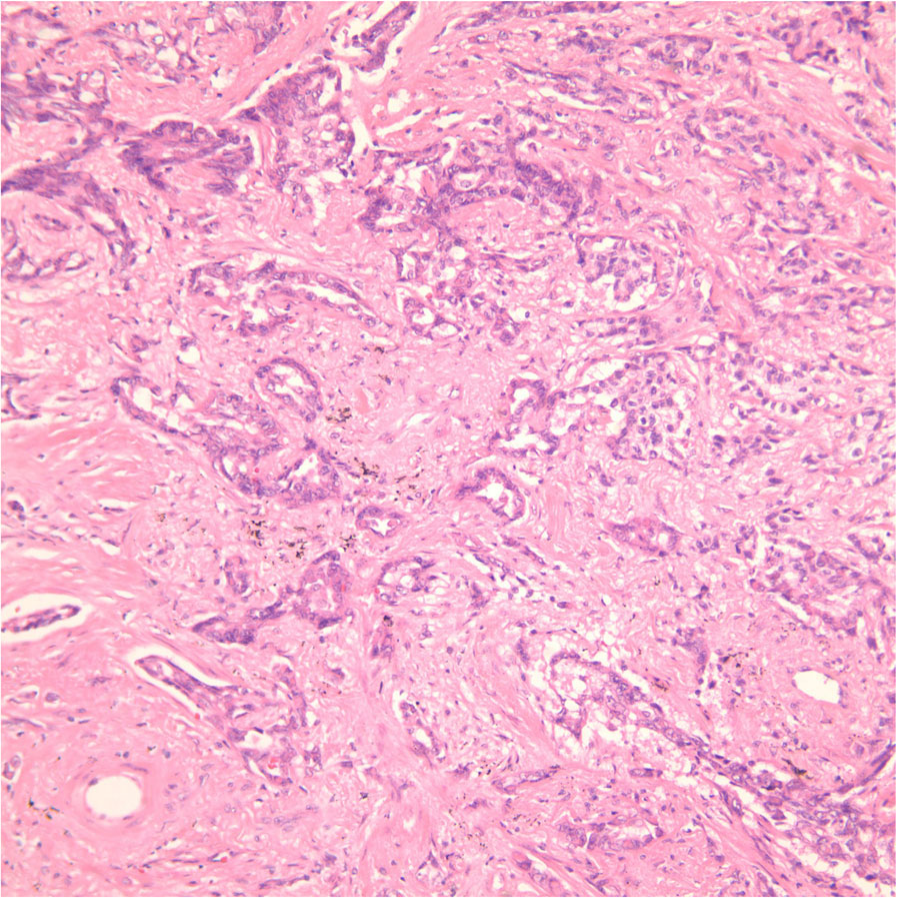
H&E stain of the patient's lung adenocarcinoma specimen under 20x magnification

**Fig. 2:**
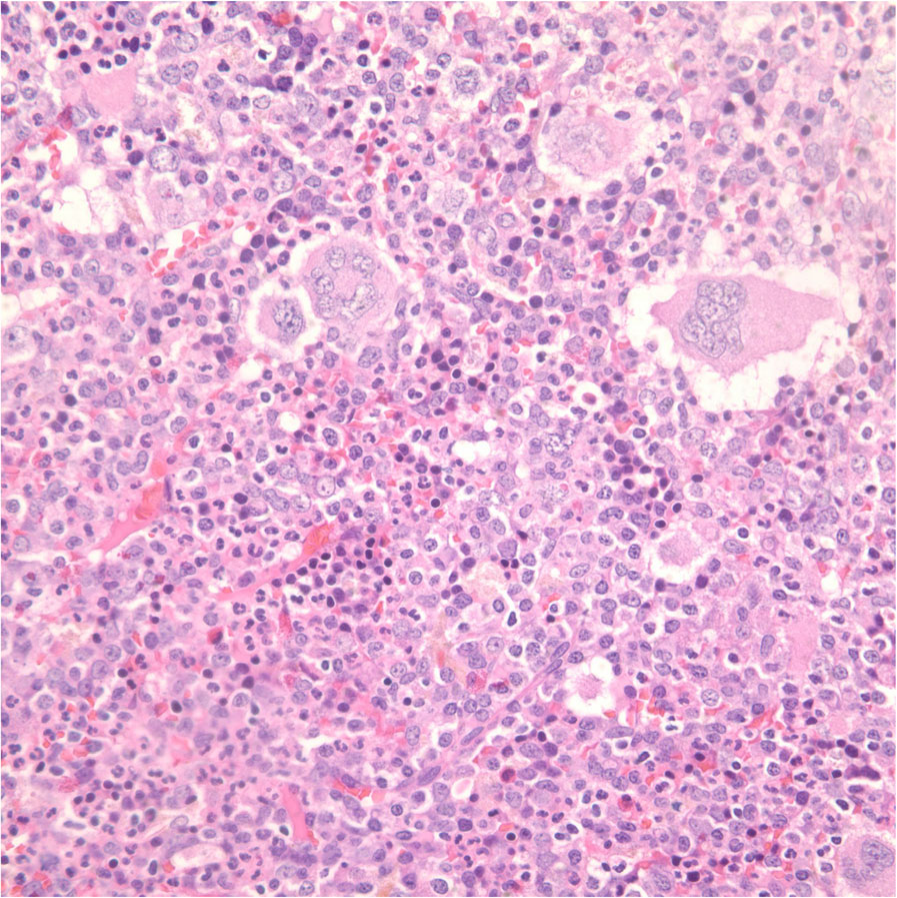
H&E stain of the patient's bone marrow biopsy consistent with MPN under 40x magnification

A 68-year-old Asian male has significant medical history of hypertension, chronic obstructive pulmonary disease, coronary artery disease requiring percutaneous coronary intervention, right lower extremity deep venous thrombosis/pulmonary embolism and peripheral vascular disease. The patient was a former long-time smoker but quitted cigarette smoking about 20 years ago. His family history was non-contributory. In 2009, patient was diagnosed with stage IA adenocarcinoma of the lung (Fig. 1), and he underwent a video-assisted thoracoscopic left upper lobe segmentectomy. At the same time, he was noted to have worsening leukocytosis and thrombocytosis. A complete blood count (CBC) at the time showed white blood cell count (WBC) 28,500/μL with a neutrophil predominance of 86.2 %, a hemoglobin of 14.7 gm/dL, and a platelet count of 737,000/mL. The patient was found to be JAK2 V617F mutation positive and BCR/ABL negative in his peripheral blood. At one point, his platelet count peaked at 1,054,000/mL. A bone marrow biopsy in October 2010 demonstrated markedly hypercellular marrow with marked megakaryocytes and mild myeloid hyperplasia consistent with MPN (Fig. 2). The patient was started on hydroxyurea with close monitoring of the blood counts. Of note, since around April 2011, patient's white blood cell monocyte percentage persistently ranged between 20-40 % and the absolute monocyte count ranged between 2,000/μL and 5,000/μL, meeting the criteria for CMML [3]. His thrombocytosis had markedly improved by that time.

**Fig. 3:**
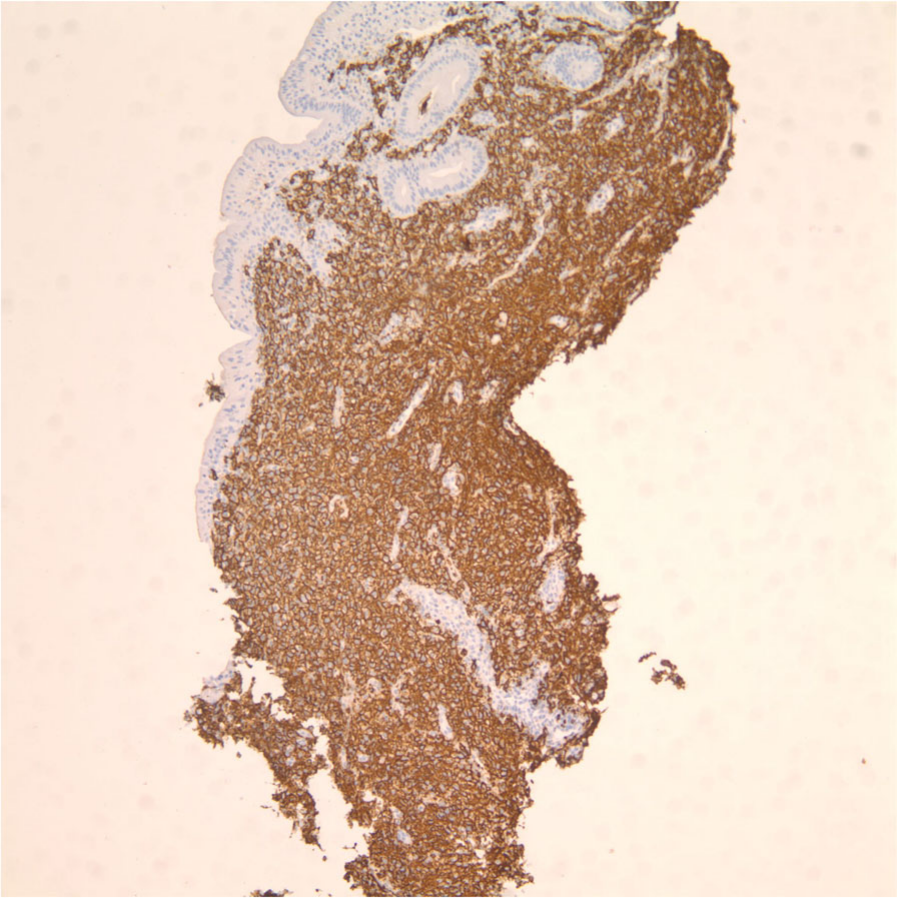
CD20 immunostain of the patient's small bowel biopsy consistent with DLBCL

In December 2013, the patient was admitted for proximal small bowel obstruction after presenting with multiple episodes of bloody bowel movements and abdominal pain. An esophagogastroduodenoscopy revealed erythematous and thickened duodenal folds. Biopsy was consistent with DLBCL (Fig. 3) and patient was staged as stage IE. Bone marrow biopsy in January 2014 did not show evidence of lymphoma but did show hypercellular marrow with myeloid hyperplasia with left shift and trilineage dyspoiesis and increased kappa restricted clonal plasma cells by flow cytometry (1 %). Given this new lymphoma diagnosis, hydroxyurea was held and chemotherapy with R-CHOP (rituximab, cyclophosphamide, doxorubicin, vincristine, prednisone) for 6 cycles were administered and completed in May 2014. His course was complicated by respiratory symptoms (coughing, shortness of breath) with pulmonary infiltrates and hilar/mediastinal lymphadenopathy noted on imaging and positive acid-fast bacillus culture for mycobacterium avium intracellulare (MAI). Fine-needle aspiration of lymph nodes showed reactive bronchial cells.

**Fig. 4:**
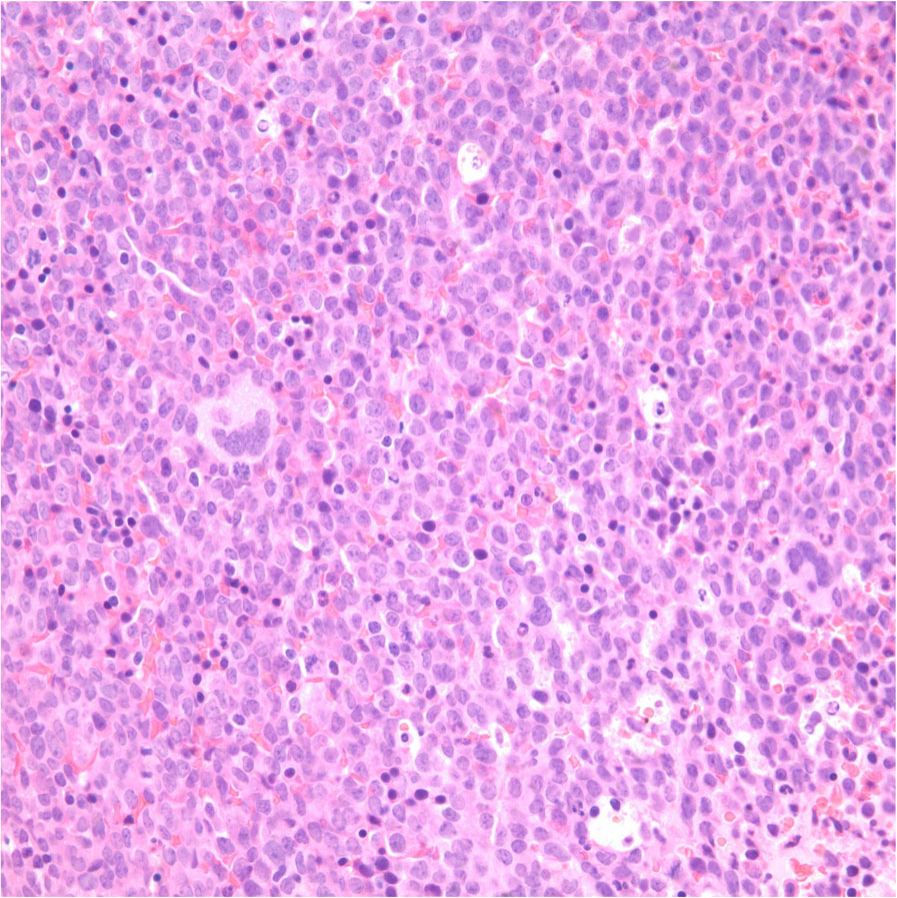
H&E stain of the patient's bone marrow biopsy consistent with AML-M5 under 40x magnification

In September 2014, the patient was noted to have a WBC of 57,800/μL (manual differentiation showing 68 % neutrophils, 4 % bands, 3 % metameylocytes, 3 % myelocytes, 2 % lymphocytes, 8 % promonocytes, 10 % monocytes, 2 % basophils), a hemoglobin of 11.8 gm/dL, and a platelet count of 243,000/mL. Given these findings, a bone marrow biopsy was performed, which demonstrated hypercellularity approaching 100 %. There was a prominent population of mature and immature/atypical monocytes comprising 77 % of analyzed WBC. These cells uniformly expressed CD11c, CD13, CD33 and CD64, with partial aberrant CD56 and variable HLA-DR consistent with left-shifted/dyspoietic maturation. These findings were consistent with acute myeloid leukemia with monocytic differentiation (AML-M5) (Fig. 4). CD34 positive myeloblasts comprised 1-2 % of cells. There was no evidence of lymphoma. Cytogenetics showed 2 of 22 metaphases had translocation of chromosome 4 and 11, loss of chromosome 9, and an additional copy of chromosome 11. Mixed lineage leukemia (MLL) gene rearrangement was noted in 57.5 % of cells. Nucleophosmin1 (NPM1) and FMS-like tyrosine kinase-3 (FLT-3) mutations were not sent as the cytogenetics placed him in the high risk AML category.

The patient was subsequently admitted in September 2014 for initiation of induction chemotherapy and considerations for allogeneic stem cell transplantation. At the time of admission, he reported night sweats and nonspecific weight loss for a few weeks. He was started on standard "7 + 3" induction chemotherapy with idarubicin 12 mg/m2 on days 1-3 and cytarabine 100 mg/m2 on days 1-7. His course was complicated by tumor lysis syndrome, cytopenias, and elevated liver enzymes. A repeat bone marrow biopsy after induction therapy in October 2014 was performed, which unfortunately showed hypercellular marrow 90 % with 13 % promonocytes/monoblasts, consistent with residual acute myeloid leukemia with monocytic differentiation. Between November and December 2014, patient had received 2 cycles of decitabine while a search for a match-unrelated donor was going on.

In January 2015, the patient developed symptoms of hyperviscosity from thrombocytosis with a platelet count greater than two million. He underwent thrombocytapheresis with an improvement in platelet count and symptoms. In March 2015, patient was admitted again with thrombocytosis to platelet count over two million. He was started on hydroxyurea to control his counts, then received haploidentical allogeneic stem cell transplant from his daughter with fludarabine/melphalan/anti-thymocyte globulin conditioning. His course was complicated by neutropenic sepsis, acute kidney and liver injuries, gastrointestinal bleed, atrial fibrillation, cardiomyopathy, volume overload and skin graft-versus-host disease. In July 2015, repeat engraftment studies showed only 9 % donor engraftment. His subsequent decreasing blood counts and monocytosis confirmed on flow cytometry were also consistent with leukemia relapse. The patient expired in September 2015 while he was on best supportive care alone.

## Discussion

MPN's (previously chronic myeloproliferative diseases) are characterized by effective clonal myeloproliferation (for example, peripheral blood granulocytosis, thrombocytosis or erythrocytosis) in the absense of dyserythropoiesis, granulocytic dysplasia or monocytosis. Based on the 2008 World Health Organization Classification Scheme, MPN's are further subdivided into "classical" (which includes chronic myelogenous leukemia (CML), polycythemia vera (PV), essential thrombocythemia (ET), primary myelofibrosis (PMF)) and "atypical" subgroups. The patient presented here met the criteria for ET, which is characterized by megakaryocyte proliferation with large and mature morphology associated with thrombocytosis in the absence of reactive thrombocytosis. Demonstration of JAK2 V617F mutation or other clonal marker is often helpful in making the diagnosis [4, 5].

As a group, MPN's have been associated with disease transformation to myelodysplasias (MDS) and/or acute myeloid leukemia (AML). The reported incidence of AML in patients with MPN's varies typically between 5-10 % depending on the specific type of MPN after 10 years of observation [6, 7]. This risk seems to be related to the underlying disease mechanism itself, but can also be due to therapy for disease treatment. Population-based data from Sweden showed that patients with MPN's who received radioactive phosphorus (P(32)) greater than 1,000 MBq and alkylating agents greater than 1 gram had a 4.6-fold (95 % CI, 2.1-9.8; *P* = 0.002) and 3.4-fold (95 % CI, 1.1-10.6; *P* = 0.015) increased risk of AML/MDS, respectively. Of note, 41 (25 %) of 162 patients with MPN's with AML/MDS development were never exposed to alkylating agents, P32 or hydroxyurea, suggesting a major role in non-treatment related factors [8].

Recent studies have also demonstrated an association between MPN's and LPN's. An Italian study reported that patients with MPN's had a 3.44-fold increased risk of developing LPN's compared with the general population, ranging from 2.86 for plasma cell disorders, 3.44 for non-Hodgkin's lymphoma, to 12.42 for chronic lymphocytic leukemia. The risk of developing non-Hodgkin's lymphoma was substantially increased to 5.71-fold in the presence of a JAK2 V617F mutation [9].

The patient was initially diagnosed with a JAK2 positive MPN, but subsequently developed monocytosis while his thrombocytosis improved. He met the World Health Organization criteria for CMML, which is defined as having > 10 % of the entire white blood cell differential, an absolute monocyte count >1,000/μL, absent BCR-ABL1 fusion gene, < 20 % myeloblasts, monoblasts and promonocytes, and dysplasia in one or more myeloid lineages [3]. CMML has both myeloproliferative and myelodysplastic features, and therefore is classified as a myelodysplastic syndrome/myeloproliferative neoplasm (MDS/MPN) [10]. The median age at diagnosis is around 70 years of age, with a 2:1 male predominance [11]. The pathogenesis of CMML is also not entirely clear. Current approaches suggested that there might be preferred order of somatic genetic mutations: TET2 or ASXL1 first, SRSF2 or an alternative splice gene second, then a signaling gene mutation inducing GM-CSF hypersensitivity and myeloproliferation in 35-40 % of cases [12].

The patient subsequently developed AML-M5. Cytogenetic studies demonstrated translocation (4;11)(q21;q24), monosomy 9 and trisomy 11. MLL gene rearrangement, which involves the chromosome band 11q23, was noted in 57.5 % of the abnormal myeloid cells. In general, chromosome abnormalities of the same types seen in primary AML are also seen in therapy-related AML/MDS [13]. It has been described in the literature that deletions or loss of chromosome arm 5q or 7q or loss of the entire chromosome (5q-/-5 and 7q-/-7) can be seen in AML/MDS after treatment with alkylating agents, whereas balanced aberrations with a rearrangement are characteristic of that related to topoisomerase II inhibitors. These balanced chromosomal aberrations include the MLL, AML1/CBFB, RARA, or NUP98 genes [14]. The MLL gene is highly conserved among species and is associated with "mixed-lineage" leukemia, where blast cells display both myeloid and lymphoid characteristics. While myeloid and lymphoid phenotypes are evenly distributed among de novo leukemia cases with these abnormalities, the vast majority (>90 %) of therapy-related AML are associated with the myeloid phenotype. Topoisomerase II inhibitors, such as the anthracyclines (e.g., doxorubicin), mitoxantrone and dactinomycin have been associated with MLL gene rearrangement, which typically has a latency period of median 30-34 months [15]. It should be noted that the MLL gene rearrangement itself cannot definitively rule out de novo AML. In one study, the incidence of AML with MLL gene rearrangement was noted to be 9.4 % in therapy-related AML vs. 2.6 % in de novo AML (P < 0.001) [16]. Given our patient's long history of MPN/CMML and his AML developing within 8 months following the use of doxorubicin with a distinct cytogenetic profile and MLL gene rearrangement, this is more suggestive of AML related to either MPN/CMML, although prior anthracycline use may play a role as well.

As noted, our patient was discovered to have a JAK2 V617F mutation while undergoing workup for MPN/CMML. The JAK2 V617F mutation has long been described as dominant gain-of-function mutation that contributes to the expansion of MPN clones. It is not only important in the diagnosis of MPN's, but is also associated with a significantly longer duration of disease and a higher rate of complications [17]. While this mutation is present in the majority of patients with MPN's, it has also been seen in the peripheral blood of healthy donors, with the percentage quoted to range between 1-10 % depending on the assay used [18, 19]. The significance of the mutation in this setting is unclear. However, in a Danish study, out of 10,507 participants were screened for the presence of JAK2 V617F and followed for up to 17.6 years after blood sampling. Prevalence of the mutation in this predominantly Caucasian population was 0.2 % (*n* = 18). Participants with the mutation were associated with increased mortality, corresponding to a multifactorially adjusted hazard ratio of 3.0 (95 % CI, 1.9-4.9). The multifactorially adjusted hazard ratios for any cancer and hematologic cancer were 3.7 (95 % CI, 1.7-8.0) and 58 (95 % CI, 13-261), respectively [2]. Thus, the results of this study suggests that the presence of the JAK2 V617F mutation may be a marker for increased risk of developing future malignancies and increased mortality.

## Conclusion

In this report, we presented a case in which a patient with a JAK2 V617F mutation developed four different hematologic and non-hematologic neoplastic processes: lung adenocarcinoma, MPN/CMML, DLBCL, and AML-M5. While the causality between JAK2 mutation and the various malignancies cannot be established in this case, we believe that this is not a mere coincidence and that the presence of JAK2 mutation is associated with the subsequent development of both hematologic and non-hematologic malignancies [2]. Identification of downstream markers of JAK2 can potentially confirm causality, although this was not performed as the extra information would not have changed management at the time. During the past decade, cancer-related gene mutation panels have been increasingly used, with some mutations yielding potential diagnostic, prognostic and therapeutic information in various malignancies [20]. The case presented here is one good example of how the presence of an activating mutation may provide insight into the subsequent development of neoplastic processes.

Based on data from the North American Association of Central Cancer Registries (NAACCR), the average annual age-adjusted incidence rates of MDS, MPN and CMML are 3.3, 2.1 and 0.3 per 100,000 people. Based on follow up data through 2004 from the Surveillance, Epidemiology, and End Results (SEER) Program, the 3-year relative survival (comparing observed survival with expected survival from a set of people with the same characteristics as the patient cohort) for MDS, MPN and CMML are 45 %, 80 % and 21 % respectively [21]. Given these statistics, there is a need for not only early recognition of these disease entities, but also improvement in therapeutics for this patient population. Further research is necessary to determine the relationships among these distinct yet closely related hematologic disorders and to evaluate the significance of the JAK2 V617F mutation in the development of both hematologic and non-hematologic malignancies.

## References

[1] Sait SN, Claydon MA, Conroy JM, Nowak NJ, Barcos M, Baer MR (2007). Translocation (4;11)(p12;q23) with rearrangement of FRYL and MLL in therapy-related acute myeloid leukemia. Cancer Genet Cytogenet.

[2] Nielsen C, Birgens HS, Nordestgaard Bø G, Kjær L, Bojesen SE (2011). The JAK2 V617F somatic mutation, mortality and cancer risk in the general population. Haematologica.

[3] Swerdlow SH, Campo E, Harris NL, Jaffe ES, Pileri SA, Stein H, Thiele J, Vardiman JW (2008). WHO Classification of Tumours of Haematopoietic and Lymphoid Tissues.

[4] Tefferi A, Vardiman JW (2008). Classification and diagnosis of myeloproliferative neoplasms: the 2008 World Health Organization criteria and point-of-care diagnostic algorithms. Leukemia.

[5] Tefferi A, Thiele J, Vardiman JW (2009). The 2008 World Health Organization classification system for myeloproliferative neoplasms: order out of chaos. Cancer.

[6] Barbui T (2004). The leukemia controversy in myeloproliferative disorders: is it a natural progression of disease, a secondary sequela of therapy, or a combination of both?. Semin Hematol.

[7] Cervantes F, Alvarez-Larran A, Talarn C, Gomez M, Montserrat E (2002). Myelofibrosis with myeloid metaplasia following essential thrombocythaemia: actuarial probability, presenting characteristics and evolution in a series of 195 patients. Br J Haematol.

[8] Bjorkholm M, Derolf AR, Hultcrantz M, Kristinsson SY, Ekstrand C, Goldin LR, Andreasson B, Birgegard G, Linder O, Malm C (2011). Treatment-related risk factors for transformation to acute myeloid leukemia and myelodysplastic syndromes in myeloproliferative neoplasms. J Clin Oncol.

[9] Vannucchi AM, Masala G, Antonioli E, Chiara Susini M, Guglielmelli P, Pieri L, Maggi L, Caini S, Palli D, Bogani C (2009). Increased risk of lymphoid neoplasms in patients with Philadelphia chromosome-negative myeloproliferative neoplasms. Cancer Epidemiol Biomarkers Prev.

[10] Orazi A, Germing U (2008). The myelodysplastic/myeloproliferative neoplasms: myeloproliferative diseases with dysplastic features. Leukemia.

[11] Parikh SA, Tefferi A (2012). Chronic myelomonocytic leukemia: 2012 update on diagnosis, risk stratification, and management. Am J Hematol.

[12] Itzykson R, Solary E (2013). An evolutionary perspective on chronic myelomonocytic leukemia. Leukemia.

[13] Pedersen-Bjergaard J (2005). Insights into leukemogenesis from therapy-related leukemia. N Engl J Med.

[14] Pedersen-Bjergaard J, Rowley JD (1994). The balanced and the unbalanced chromosome aberrations of acute myeloid leukemia may develop in different ways and may contribute differently to malignant transformation. Blood.

[15] Pui CH, Relling MV (2000). Topoisomerase II inhibitor-related acute myeloid leukaemia. Br J Haematol.

[16] Schoch C, Schnittger S, Klaus M, Kern W, Hiddemann W, Haferlach T (2003). AML with 11q23/MLL abnormalities as defined by the WHO classification: incidence, partner chromosomes, FAB subtype, age distribution, and prognostic impact in an unselected series of 1897 cytogenetically analyzed AML cases. Blood.

[17] Kralovics R, Passamonti F, Buser AS, Teo SS, Tiedt R, Passweg JR, Tichelli A, Cazzola M, Skoda RC (2005). A gain-of-function mutation of JAK2 in myeloproliferative disorders. N Engl J Med.

[18] Sidon P, El Housni H, Dessars B, Heimann P (2006). The JAK2V617F mutation is detectable at very low level in peripheral blood of healthy donors. Leukemia.

[19] Xu X, Zhang Q, Luo J, Xing S, Li Q, Krantz SB, Fu X, Zhao ZJ (2007). JAK2(V617F): Prevalence in a large Chinese hospital population. Blood.

[20] Meldrum C, Doyle MA, Tothill RW (2011). Next-Generation Sequencing for Cancer Diagnostics: a Practical Perspective. Clin Biochem Rev.

[21] Rollison DE, Howlader N, Smith MT, Strom SS, Merritt WD, Ries LA, Edwards BK, List AF (2008). Epidemiology of myelodysplastic syndromes and chronic myeloproliferative disorders in the United States, 2001-2004, using data from the NAACCR and SEER programs. Blood.

